# Ramipril-Induced Isolated Small Bowel Angioedema: A Case Report

**DOI:** 10.7759/cureus.83987

**Published:** 2025-05-12

**Authors:** Mohamad Al Ayoubi, Fatima I Hsayan, Tasneem M Freijeh, Diana Jabbour, Antoine S Geagea

**Affiliations:** 1 Gastroenterology, Lebanese University Faculty of Medicine Sciences, Hadath, LBN; 2 Internal Medicine, Lebanese University Faculty of Medicine Sciences, Hadath, LBN; 3 Radiology, Lebanese University Faculty of Medicine Sciences, Hadath, LBN; 4 Gastroenterology and Hepatology, Lebanese Hospital Geitaoui - University Medical Center, Beirut, LBN; 5 Gastroenterology and Hepatology, Lebanese University Faculty of Medicine Sciences, Hadath, LBN

**Keywords:** ace inhibitor, ace inhibitor induced angioedema, drug-induced angioedema, rare side effect, small bowel angioedema

## Abstract

Isolated small bowel angioedema is an uncommon adverse reaction to angiotensin-converting enzyme (ACE) inhibitors, predominantly affecting females. The nonspecific clinical presentation, along with the lack of definitive diagnostic laboratory and radiological findings, makes the diagnosis particularly challenging.

We present the case of a 63-year-old female patient who developed diffuse abdominal pain and nausea just seven days after starting ramipril. The diagnosis of ACE inhibitor-induced small bowel angioedema was confirmed by a computed tomography (CT) scan of the abdomen and the pelvis, which revealed circumferential wall thickening and submucosal edema involving multiple jejunal loops. These findings were consistent with angioedema. The condition was successfully managed by discontinuing the medication, and the patient showed rapid clinical improvement within 48 hours of stopping ramipril.

To our knowledge, this is only the second reported case of ramipril-induced isolated small bowel angioedema in the literature.

## Introduction

Angioedema presents as pale, non-itchy, well-defined, and non-pitting edema that involves the skin, subcutaneous tissue, or submucosa. It is a rare adverse effect of angiotensin-converting enzyme inhibitors (ACEIs) and affects 0.1% to 2.2% of individuals receiving ACEI therapy [1].

ACEI-induced angioedema typically affects the face, tongue, lips, and upper respiratory tract. Isolated small bowel angioedema is an exceptionally rare complication of ACEI therapy [2]. Angioedema resulting from ACEIs can manifest either soon after treatment initiation or after years of continued use. It is independent of dosage. By inhibiting the breakdown of bradykinin, ACEIs lead to its accumulation, particularly in the upper respiratory and gastrointestinal tracts. Elevated bradykinin levels contribute to vasodilation and enhanced vascular permeability [3].

Small bowel angioedema due to ACEIs often goes clinically undiagnosed because its symptoms and radiographic presentation closely resemble those of several other conditions, including small bowel ischemia, enteritis, lymphoma, vasculitis, C1 esterase deficiency, and Crohn’s disease [2]. Patients usually present with abdominal pain of variable duration [4]. To establish the diagnosis, a contrast-enhanced CT of the abdomen and pelvis is frequently obtained, showing findings such as diffuse mural and mucosal thickening of the small bowel, prominent mucosal enhancement, fluid-filled distention of the small bowel, mesenteric vascular engorgement, and the presence of ascites, all of which suggest small bowel angioedema [3].

We present the case of a 63-year-old female patient who complained of diffuse abdominal pain one week after starting ramipril for newly-diagnosed hypertension.

## Case presentation

A 63-year-old female patient presented to the clinic with a day's history of diffuse abdominal pain and mild nausea. There was no diarrhea or vomiting, and bowel movements were normal.

The patient had no known food or drug allergies, was a smoker, and did not consume alcohol. She had a known history of hypertension, for which she had been taking bisoprolol 5 mg daily. Ramipril 5 mg daily was added to her regimen one week ago. She also had dyslipidemia, which was managed with rosuvastatin 20 mg daily. Her surgical history was significant for a cesarean section.

Physical examination revealed diffuse abdominal tenderness without any localized pain, and her vital signs were within normal limits.

Laboratory evaluation showed a mild elevation in C-reactive protein (CRP), with a normal complete blood count (CBC), basic metabolic panel, liver enzymes, and lipase, as shown in Table 1.

**Table 1:** Key laboratory test results

Test	Result	Reference range
Leukocyte count (/mm^3^)	8,510	4,800–10,800
Neutrophils (%)	58	60–70
Hemoglobin (g/dL)	14.4	12–16
Platelet count (/mm^3^)	216,000	130,000–400,000
Creatinine (mg/dL)	0.62	0.44–1.0
Sodium (mmol/L)	138	135–145
Potassium (mmol/L)	4.16	3.5–5.0
Chloride (mmol/L)	101.6	98–106
C-reactive protein (mg/L)	17.9	0–6
Aspartate aminotransferase (AST; U/L)	14	10–40
Alanine aminotransferase (ALT; U/L)	15	7–56
Gamma-Glutamyl Transferase (GGT; U/L)	12	9–48
Alkaline phosphatase (ALP; U/L)	48	44–147
Lipase (U/L)	25	13–60

**Figure 1:**
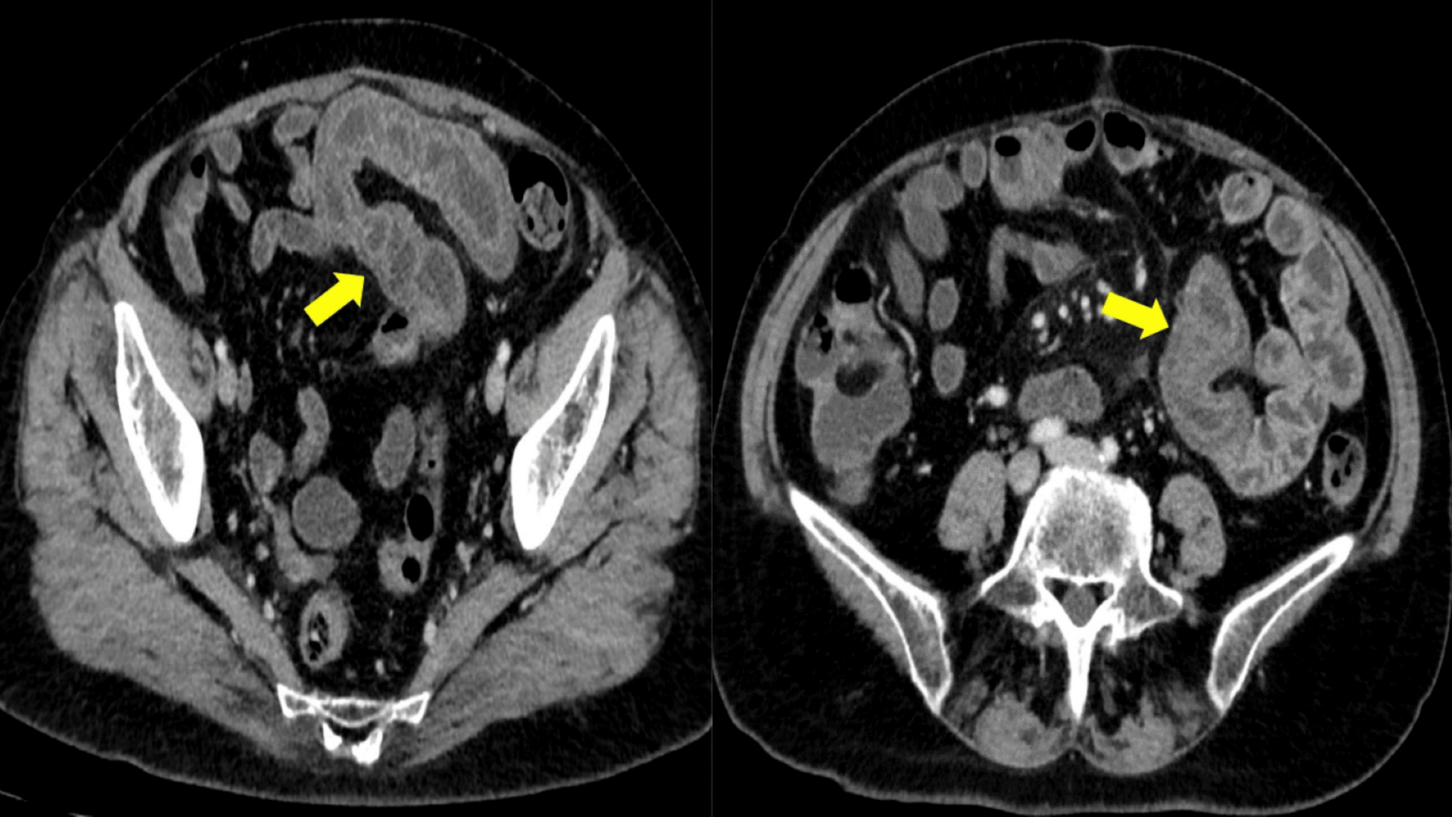
Concentric submucosal thickening of a long small bowel segment with mural stratification and hyperemia (yellow arrow) Straightening of bowel loops also noted.

A CT scan of the abdomen and pelvis with IV contrast was performed, and it revealed circumferential wall thickening and submucosal edema involving multiple jejunal loops, findings consistent with angioedema (Figure 1).

**Figure 2:**
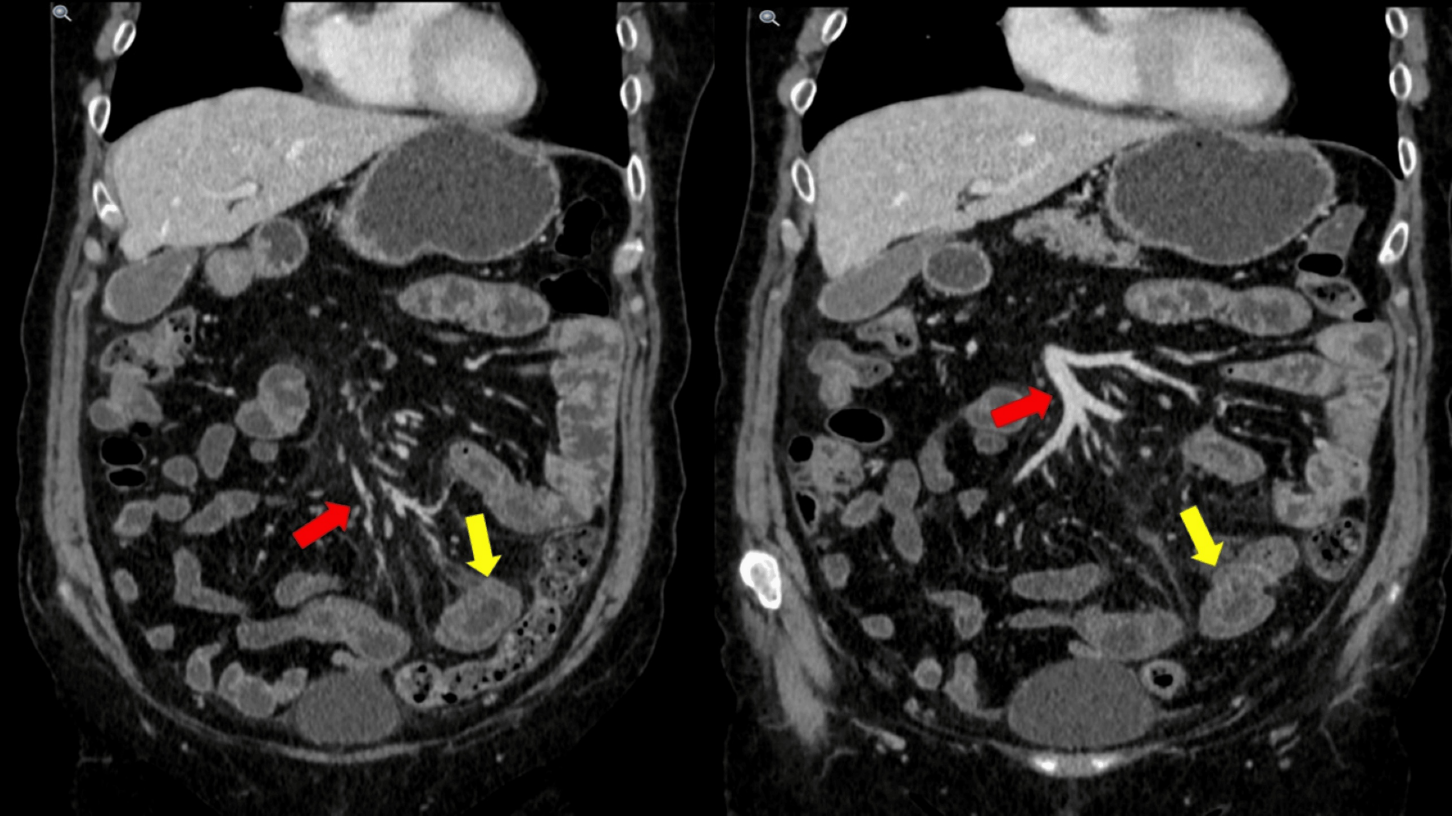
Mesenteric edema and congestion (red arrow) with no associated lymphadenopathy or bowel obstruction, and typical target sign (yellow arrow) affecting a segment of the jejunum noted

There was no evidence of bowel obstruction or perforation, and the mesenteric vasculature appeared patent. A trace amount of free fluid was present, and the colon was normally distended. Additionally, mesenteric congestion was noted (Figure 2).

Based on the findings of the imaging studies and her clinical history, the patient was diagnosed with ACEI-induced small bowel angioedema. Ramipril was immediately discontinued, and the patient showed significant clinical improvement within 48 hours of discontinuation.

## Discussion

ACEIs are widely prescribed for the management of hypertension and various other cardiovascular conditions [5]. They are a major cause of drug-induced angioedema in the United States, accounting for 20% to 40% of all angioedema-related emergency department visits annually [6]. Angioedema is characterized by pale, non-itchy, well-defined, non-pitting edema involving the skin, subcutaneous tissue, or submucosa, and it affects 0.1% to 2.2% of individuals treated with ACEIs [1].

ACEI-induced angioedema typically involves the tongue, lips, face, and upper airways. Isolated small bowel involvement is extremely rare. Among reported cases of isolated small bowel angioedema, 85.3% occurred in females, with an average age of 49.5 years [2]. Lisinopril is the most commonly reported culprit among all ACEIs, responsible for 56% of cases [7].

The duration of ACEI treatment at the onset of angioedema ranges from one day to 8 years, with a median duration of six months [8]. The condition is not related to the dose of the ACEI taken [2]. Angiotensin-converting enzyme (ACE) is structurally similar to kininase II, an enzyme responsible for the degradation of bradykinin into inactive peptides. Inhibition of ACE prevents the breakdown of bradykinin, resulting in its elevated levels. Increased bradykinin levels lead to vasodilation and enhanced permeability of postcapillary venules, allowing plasma to extravasate into the submucosal tissue, thereby causing angioedema [2]. Enzymes such as aminopeptidase P (APP) and dipeptidyl peptidase IV (DPP-IV) are involved in breaking down bradykinin and substance P. Reduced activity of APP and DPP-IV has been reported in multiple case-control studies of patients experiencing ACEI-related angioedema. Additionally, estrogen promotes bradykinin expression, which may contribute to the higher incidence observed in females [1].

Hereditary angioedema is characterized by low levels of C4 and C1 esterase inhibitors. Therefore, in cases of isolated small bowel angioedema, it is essential to rule out C1 esterase inhibitor deficiency by measuring C4 and C1 inhibitor antigen levels, as well as performing functional assays for C1 esterase inhibitor activity [2].

Patients with small bowel angioedema typically present with abdominal pain, nausea, and vomiting. Less commonly, diarrhea and/or ascites may occur [8]. Symptoms usually resolve spontaneously within 24 to 36 hours, with or without discontinuation of the ACEI. Laboratory results are typically normal or show only a mild elevation in white blood cell count. CT imaging plays a crucial role in the diagnosis and commonly reveals circumferential small bowel wall thickening (which may be segmental), mural stratification (the “target sign”), elongation or straightening of the bowel loops, mesenteric vessel engorgement, mesenteric edema with or without ascites, and absence of vascular compromise or lymphadenopathy [9].

In a review by Scheirey et al., 20 patients experienced 23 separate episodes of ACEI-induced small bowel angioedema, all evaluated by CT imaging. The scans showed mildly dilated small bowel loops, with an average diameter of 2.9 cm, and bowel wall thickening averaging 1.3 cm. A characteristic “small bowel target sign” was noted in 16 of the 20 multidetector CT scans. The affected bowel segments were most frequently located in the jejunum alone (10 out of 23 cases), followed by the ileum alone (seven out of 23), and both jejunum and ileum (six out of 23) [7].

The differential diagnosis for small bowel angioedema includes inflammatory bowel disease, infectious enteritis, bowel ischemia, vasculitis, and mechanical obstruction [10]. Diagnosis is typically based on a combination of the patient’s medication history and CT findings [2]. To make the diagnosis, physicians must maintain a high index of suspicion [11].

The standard treatment for ACEI-induced angioedema is immediate discontinuation of the offending medication. Symptoms typically resolve within 24 to 72 hours, and patients should be observed in a hospital setting for at least 24 hours. Controlled studies have not demonstrated consistent efficacy for adjunctive treatments such as antihistamines, subcutaneous epinephrine, or corticosteroids. Patients who have experienced ACEI-induced angioedema should avoid ACEI permanently, and the medication should be documented as a drug allergy [1].

To date, only one case of ramipril-induced small bowel angioedema has been reported [10], making our case the second. This highlights the need for increased awareness of the potential gastrointestinal side effects of ramipril.

## Conclusions

We describe a patient who developed abdominal pain and nausea, later diagnosed as small bowel angioedema resulting from recently started ACEI therapy. A comprehensive medication history, along with typical CT scan findings, plays a crucial role in making the correct diagnosis and preventing unnecessary medical therapy or invasive interventions, such as surgery. Discontinuing the ACEI and closely monitoring the patient generally leads to a swift resolution of symptoms. It is vital to avoid reintroducing ACEIs in such patients to prevent the recurrence of angioedema.
